# TRIM: Simultaneous Thermometry, Ranging, and Imaging via a Monolithic Metalens

**DOI:** 10.1002/advs.75776

**Published:** 2026-05-28

**Authors:** Man Yuan, Yuqing Zhang, Wangzhe Zhou, Zhaojian Zhang, Xiaoyun He, Xinpeng Jiang, Yiyi Li, Xin He, Jiagui Wu, Yuanmu Yang, Junbo Yang

**Affiliations:** ^1^ College of Science National University of Defense Technology Changsha China; ^2^ School of Physical Science and Technology Southwest University Chongqing China; ^3^ State Key Laboratory of Precision Measurement Technology and Instruments Department of Precision Instrument Tsinghua University Beijing China

**Keywords:** long‐wave infrared (LWIR), metalens, monolithic integration, multidimensional sensing, passive ranging, ratiometric thermometry

## Abstract

While metasurfaces offer a pathway beyond the discrete architectures of conventional LWIR systems, physically fusing high‐precision thermometry and passive ranging onto a single metalens remains a formidable challenge. Here, we demonstrate a monolithic, dual‐focus metalens capable of simultaneous multidimensional sensing. By multiplexing two distinct focal lengths into a single metasurface, the system enables the parallel acquisition of spectral and spatial information. For thermal sensing, we implement a dual‐band (9.6 and 10.6 µm) ratiometric method that effectively mitigates target emissivity uncertainty under the gray‐body assumption. This approach yields a mean absolute temperature error of 13.58°C (∼3% relative error) and exhibits exceptional linearity (R^2^ > 0.916) across 100°C–450°C. Concurrently, the dual‐focus architecture facilitates passive depth acquisition by capturing differential defocus signatures. A depth retrieval model based on the normalized defocus response establishes a robust linear relationship with inverse depth (R^2^ = 0.916), with measurement deviations primarily confined within the 95% confidence interval across the 15–25 cm range. Our approach opens a pathway for transitioning from discrete sensors to monolithic, chip‐scale intelligent perception platforms, enhancing simultaneous thermometry and ranging for remote targets without compromising diffraction‐limited imaging performance.

## Abstract 2

A monolithic dual‐focus LWIR metalens is demonstrated for simultaneous emissivity‐independent thermometry and passive ranging. By multiplexing focal lengths, the system achieves a 3% temperature error across 100°C–450°C and robust depth retrieval (R^2^ = 0.916) within 15–25 cm. This work facilitates a transition toward chip‐scale, multi‐parameter intelligent perception for integrated, high‐performance infrared sensing platforms.

## 1 Introduction

The long‐wave infrared (LWIR, λ = 8–14 µm) spectral range [1, 2, 3, 4] is a key atmospheric window of mid‐infrared [5, 6, 7, 8] with wide applications [9, 10, 11, 12, 13], including thermal imaging [2, 14, 15], environmental non‐contact sensing [16, 17, 18, 19], industrial non‐destructive testing [20], and security monitoring. In these scenarios, the simultaneous realization of non‐contact quantitative temperature measurement [21, 22, 23, 24, 25, 26] and distance perception [27, 28, 29, 30, 31] is crucial, as it can significantly enhance the automation and intelligent decision‐making capabilities of infrared systems [19]. Traditional optical solutions can separately handle thermal radiation imaging [5, 31] and ranging via LiDAR (Light Detection and Ranging) [20, 32, 33] or binocular vision [22]. However, the physical stacking of multiple modules results in bulky, high‐power‐consumption systems and induces challenges regarding optical axis alignment and field‐of‐view matching.

To address these challenges, metasurfaces—planar optical devices composed of subwavelength artificial building blocks—have emerged as a transformative platform, enabling a paradigm shift from conventional volumetric optics to ultrathin, interface‐based photonic systems. By precisely engineering the spatial distribution of phase, amplitude, and polarization at the subwavelength scale, metasurfaces have demonstrated remarkable capabilities across a broad range of applications, including high‐performance imaging [34, 35], multi‐channel optical communication [36, 37], structural colors [38, 39], and advanced beam shaping [40, 41]. In particular, metalenses—flat lenses based on metasurface architectures that focus light through engineered phase profiles—have achieved significant milestones in imaging systems and temperature–distance sensing technologies. For instance, Huang et al. introduced all‐silicon meta‐optics for broadband thermal imaging in the LWIR regime, enabling diffraction‐limited performance across 8–14 µm with a large field of view through inverse design and computational imaging techniques [2]. Similarly, Meng et al. advanced polarization‐insensitive, broadband achromatic metalenses tailored for mid‐to‐long‐wave infrared detectors, effectively compensating chromatic aberrations via nanostructure optimization [6]. Earlier foundational works by Khorasaninejad et al. demonstrated diffraction‐limited visible metalenses and multispectral chiral imaging capabilities [42], while Shalaginov et al. showcased reconfigurable all‐dielectric metalenses with dynamic phase tuning for versatile wavefront control [12]. These innovations, complemented by the nanophotonic LiDAR strategies presented by Kim et al. [29], collectively underscore the significant potential of metalenses. Furthermore, inspired by jumping spider eyes, Guo et al. designed a compact single‐shot metalens depth sensor that utilizes dual focal planes to simultaneously capture images with different defocus, achieving efficient passive ranging [27]. These works have advanced high‐quality imaging and singular depth perception using metalens; however, constrained by material dispersion and absorption, as well as the decoupling design of multispectral radiation and wavefront information, the synergistic extraction of thermal radiation characteristics and distance wavefront information within a single planar element has not yet been achieved.

Precision in LWIR thermometry is fundamentally limited by surface emissivity uncertainty and atmospheric attenuation (e.g., water vapor and CO_2_ absorption [8]), whereas ranging relies on resolving wavefront phase or defocus cues; achieving high accuracy in both requires coupling multispectral ratiometry with static light‐field manipulation. To address radiometric inaccuracies, dual‐band colorimetric meta‐thermal imaging has been demonstrated to reduce emissivity‐induced errors by up to 50% [21], while other approaches have leveraged LWIR atmospheric absorption combined with defocus for passive ranging [43]. The Guo et al. scheme further demonstrated that by spatially multiplexing dual‐defocus images via a metalens, depth can be efficiently decoded on a single sensor requiring <700 FLOPs/pixel(floating point operations) [27]. Similarly, Zhao et al. developed a polarization‐splitting metalens to simultaneously capture orthogonal polarization images, enhancing contrast in turbid water and estimating depth [30]. However, the dual‐defocus imaging methods in the visible and NIR band limited radiometric temperature coupling. Despite progress in these single‐parameter schemes, such as dual‐spectral ratios to mitigate emissivity issues, or defocus/polarization for depth extraction, multi‐parameter applications remain constrained by heavy computational overhead, long‐distance atmospheric interference, and a lack of monolithic integration. There is an urgent need for precise light‐field manipulation by LWIR metalens, enabling physical‐level integration of radiometric thermometry and passive ranging through radiometric spectral and wavefront information [43, 44, 45].

**FIGURE 1:**
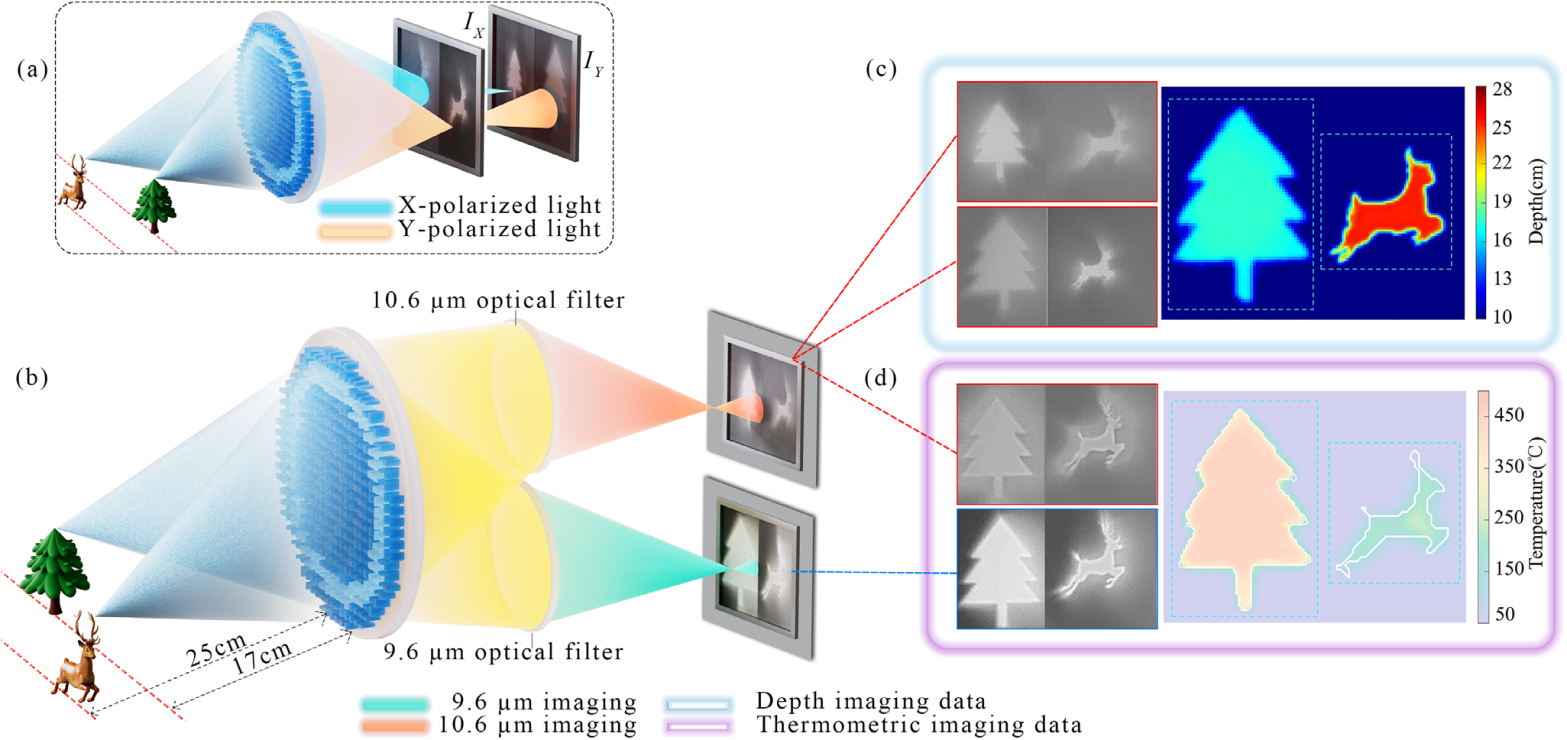
Schematic of the metalens‐based system for simultaneous imaging, thermometry, and ranging. (a) Illustration of the bifocal metalens imaging system generating image pairs with controllable defocus difference. Here, *I*_X_ and *I*_Y_ represent the defocused images formed by *X*‐ and *Y*‐polarized light, respectively. (b) Experimental setup featuring miniature deer and tree models positioned at distinct distances and temperatures as the imaging target. The deer is located 25 cm from the metalens at 250°C, whereas the tree is positioned at 17 cm at 450°C. The scene is imaged through the metalens, with narrow‐band filters centered at 9.6 and 10.6 µm inserted into the optical path. Images are acquired across various defocus levels and spectral bands. (c) Depth map of the target reconstructed using two images with different defocus levels captured at 10.6 µm via the bifocal metalens. (d) Temperature distribution map retrieved via two‐color pyrometry utilizing the corresponding images at 9.6 and 10.6 µm.

To address these challenges, this study proposes and experimentally demonstrates a LWIR dual‐focus metalens operating in the 9.6–10.6 µm band. For the first time, dual‐band colorimetric thermometry and defocus‐based ranging mechanisms are deeply integrated into a monolithic optical system, as shown in Figure 1b. Unlike traditional discrete sensors, we achieved independent wavefront manipulation of the dual‐band light field within a unified 12 mm aperture through nanostructured metalens design, synchronously generating image pairs with controllable defocus disparity in the system, as shown in Figure 1a. For passive ranging, we established a depth retrieval model centered on the normalized defocus ratio (δ*I*/∇^2^ *I*) as the core physical metric, revealing a significant, deterministic linear relationship between this metric and the target inverse depth. Experimental results demonstrate that within the 15–25 cm ranging window, the linear model achieves a coefficient of determination (*R* ^2^) of 0.916, with data points tightly clustered within the 95% confidence interval. This proves the feasibility and robustness of passive ranging using single‐frame defocus characteristics, realizing high‐precision single‐frame ranging, as shown in Figure 1c. Simultaneously, the system exploits the correlation between the dual‐band radiant intensity ratio (at 9.6 and 10.6 µm) and Planck's function to effectively circumvent the uncertainty of object surface emissivity. The algorithm was validated across a broad temperature range of 100°C–450°C. Results indicate a high degree of linearity between measured and true temperatures; at distinct depths of 17 and 19 cm, the *R* ^2^ values for the linear fits reached 0.9647 and 0.9787, respectively. The system exhibited superior thermometric precision, with a minimum mean absolute error (MAE) of only 13.58°C and a root mean square error (RMSE) controlled within 22.24°C, realizing parallel high‐precision thermometry, as shown in Figure 1d. Thus, while maintaining diffraction‐limited imaging quality, this system marks a transition in LWIR temperature and distance detection from discrete sensors to a “chip‐scale” integrated intelligent sensing platform, providing a novel technical pathway for the synchronous and precise detection of distant targets in complex environments.

## 2 Principle

### 2.1 Defocus Ranging Theory

**FIGURE 2:**
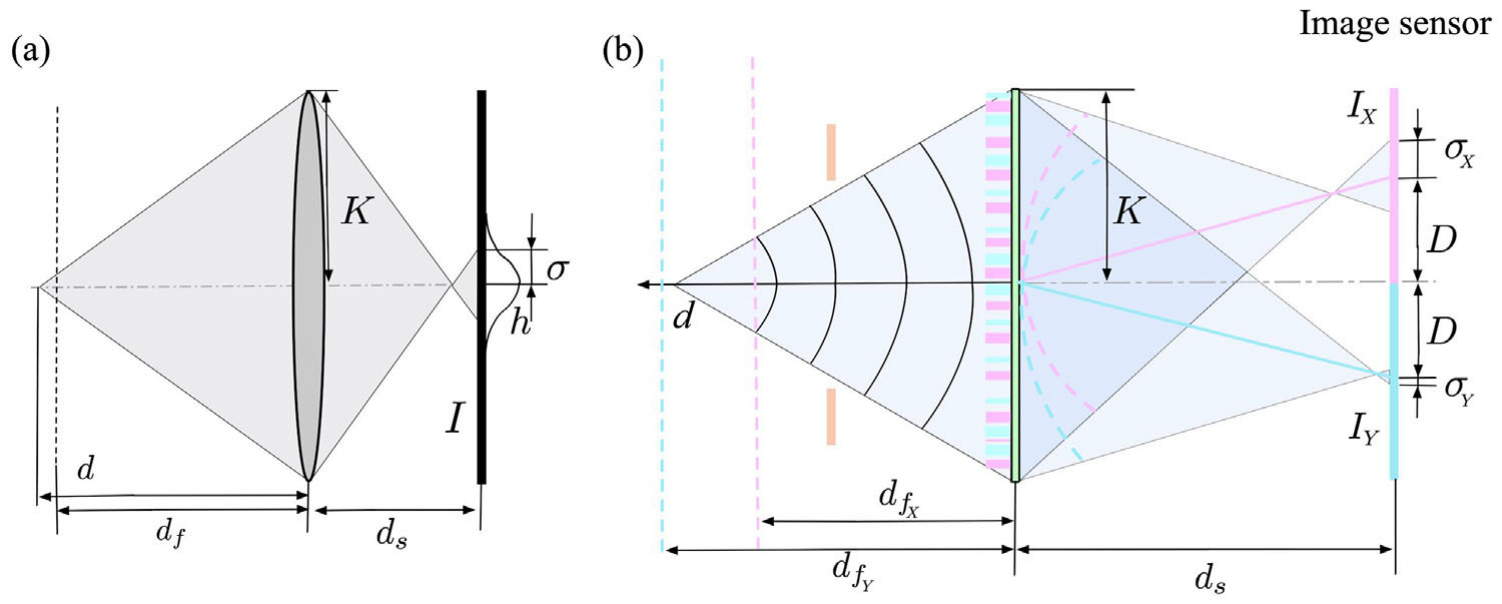
Operating principle of the bifocal metalens imaging system. (a) Conventional thin‐lens camera model. The width of the point spread function (PSF),σ, varies with the object distance *d*. Here, *d*_s_ represents the lens‐to‐sensor distance, *d*_f_ is the focal length, and *K* is the aperture radius. The solid black curve depicts a vertical cross‐section of the Gaussian PSF, *h*. (b) Bifocal metalens designed in this work. The metalens multiplexes two distinct thin‐lens phase profiles within a single aperture. For incident X‐ and Y‐polarized light, it generates two different focal lengths $d_{f_{X}}$ (red) and $d_{f_{Y}}$ (blue). By focusing off‐axis, it yields two side‐by‐side images (*I*_X_,*I*_Y_) exhibiting different PSF widths (σ_X_,σ_Y_), with their centers transversely shifted by ± *D* from the optical axis. The red and blue dashed curves adjacent to the metalens indicate the transmitted wavefronts. Due to spatial multiplexing, the resulting overall phase distribution is highly discontinuous—a complex profile not readily achievable with conventional diffractive (e.g., Fresnel) optics.

To model the bifocal imaging system, we define the ideal in‐focus scene as *f*(*x*, *y*). Consequently, the pair of defocused images as shown in Figure 2 is given by [46]:

(1) $$\begin{array}{c} I_{X}\left(x,y\right)=h_{X}\left(x,y;d\right)*f\left(x,y\right)\\ I_{Y}\left(x,y\right)=h_{Y}\left(x,y;d\right)*f\left(x,y\right) \end{array}$$

Here, *h*_X,Y_ denotes the point spread function (PSF) corresponding to the specific focal length, and *d* represents the object distance. Within the framework of geometrical optics, the PSF of the off‐axis imaging system can be approximated as a shifted Gaussian function:

(2) $$h_{X,Y}\left(x,y;d\right)=\frac{1}{2\pi \sigma_{X,Y}^{2}}\exp\left[-\frac{x^{2}+y^{2}}{2\sigma_{X,Y}^{2}}\right]$$

The relationship between the spread parameter σ_X,Y_ and the object distance is derived from the thin lens formula:

(3) $$\sigma_{X,Y}=\left[\left(\frac{1}{d_{f_{X,Y}}}-\frac{1}{d}\right)d_{s}\right]K$$

where *K* denotes the effective aperture radius of the system, and *d_s_* is the image distance. When the design satisfies the condition $d_{f_{X}}\approx d_{f_{Y}}$, a differential defocus approximation can be introduced. Defining the average focal length as $d_{f}=\left(d_{f_{X}}+d_{f_{Y}}\right)/2$ and the focal length difference as $\delta d_{f}=d_{f_{X}}-d_{f_{Y}}$, the first‐order approximation yields:

(4) $$\frac{1}{d_{f_{X,Y}}}\approx \frac{1}{d_{f}}\mp \frac{\delta d_{f}}{2d_{f}^{2}}$$

The corresponding variation in blur parameters is given by:

(5) $$\delta \sigma =\sigma_{X}-\sigma_{Y}\approx Kd_{s}\left(\frac{1}{d_{f_{X}}}-\frac{1}{d_{f_{Y}}}\right)=\frac{Kd_{s}\delta d_{f}}{d_{f}^{2}}$$

Through algebraic manipulation, a closed‐form formula for estimating depth directly from the image data is derived:

(6) $$\begin{array}{cc} d(x,y) & ={\left(\alpha +\beta \cdot \frac{F*\delta I}{F*\nabla^{2}I}\right)}^{-1}\;\\ & ={\left(\alpha +\beta \cdot \frac{F*(I_{X}\left(x,y\right)-I_{Y}\left(x,y\right))}{F*\nabla^{2}I\left(x,y\right)}\right)}^{-1} \end{array}$$

(7) $$\begin{array}{cc} & \alpha =\frac{1}{2}\left(\frac{1}{d_{f_{X}}}+\frac{1}{d_{f_{Y}}}\right)\\ & \beta =-\frac{1}{{\left(Kd_{s}\right)}^{2}}{\left(\frac{1}{d_{f_{X}}}-\frac{1}{d_{f_{Y}}}\right)}^{-1} \end{array}$$

The parameter α corresponds to the mean of the two inverse focal lengths, determining the baseline offset for depth calculation. The parameter β is inversely proportional to the sensitivity of blur variations; specifically, a smaller focal length difference yields a larger β, thereby enhancing the system's sensitivity to depth fluctuations. Defined as the normalized defocus response *G* = (*I*_X_ − *I*_Y_)/∇^2^ *I*, this ratio term in Equation 6 represents the relationship between the contrast variation caused by defocus and the curvature of the local image structure. Additionally, *F* denotes a linear filter kernel designed to suppress noise and optical artifacts, where * represents the convolution operation and ∇^2^ is the discrete Laplacian operator. The final confidence function is defined as:

(8) $$c\left(x,y\right)=f\left(|\gamma_{1}\delta I+\gamma_{2}\frac{1}{\nabla^{2}I}+\gamma_{3}|\right)$$

where $\gamma_{1,2,3}$ are confidence parameters that depend on the dimensions of the optics. This establishes a quantitative mapping from image features to estimation reliability, providing a critical uncertainty metric for depth sensing systems. The derivation strikes a balance between theoretical rigor and computational feasibility. Validated in practical implementations, it constitutes a fundamental component of depth‐from‐defocus theory.

Further mathematical derivations of higher‐order correction models, along with comparisons to our first‐order approximation, have been added to Note S1, showing that neglecting higher‐order terms has a negligible impact on the accuracy of our results.

### 2.2 Principle of Temperature Measurement Based on Planck's Law

In the LWIR regime, Planck's law can be written as:

(9) $$M_{b}\left(\lambda ,T\right)\approx \frac{2\pi hc^{2}}{\lambda^{5}}\frac{1}{\exp\left(\frac{\mathrm{hc}}{\lambda \mathrm{kT}}\right)-1}$$

where 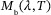 denotes the spectral radiant exitance (W · m^−3^), *T* represents the absolute temperature (K), and λ is the wavelength (fixed at 10.6 µm in this study). The physical constants include Planck's constant *h* (6.626  ×  10^−34^J·s), the speed of light *c* (3.0  ×  10^8^ *m*/*s*), and the Boltzmann constant *k* (1.38 × 10^−23^ J/K).

Real objects are typically modeled as gray bodies, with their radiative properties corrected by the emissivity, ε(λ, *T*):

(10) $$M_{\mathrm{actual}}\left(\lambda ,T\right)=\varepsilon \left(\lambda ,T\right)\cdot M_{b}\left(\lambda ,T\right)$$

This emissivity, ε(λ, *T*), is a function of surface characteristics, wavelength, temperature, and viewing angle, representing the most pivotal and complex parameter in infrared thermometry.

Consequently, in practical measurements, the detector captures the integrated radiant power within a finite optical bandwidth Δλ. For a dual‐channel system with center wavelengths λ_1_ and λ_2_, and corresponding bandwidths Δλ_1_ and Δλ_2_, the output signals I_1_(T) and I_2_(T) are expressed as:

(11) $$\begin{array}{c} I_{1}\left(T\right)=\int_{\Delta \lambda_{1}}\varepsilon \left(\lambda ,T\right)\cdot M_{b}\left(\lambda ,T\right)\cdot F_{1}\left(\lambda \right)\;d\lambda \\ I_{2}\left(T\right)=\int_{\Delta \lambda_{2}}\varepsilon \left(\lambda ,T\right)\cdot M_{b}\left(\lambda ,T\right)\cdot F_{2}\left(\lambda \right)\;d\lambda \end{array}$$

where *F*_1_(λ) and *F*_2_(λ) represent the system spectral response functions for the two respective channels. The fundamental principle of colorimetric thermometry relies on the ratio of these two signal bands, *R*(*T*), to derive the target temperature [21]. If the target can be approximated as a gray body, implying that its emissivity exhibits negligible spectral dependence, ε(λ, *T*) can be treated as a temperature‐dependent constant, ε(*T*), within the specified narrow bands Δλ_1_ andΔλ_2_. Under this assumption, the above equation simplifies to [21]:

(12) $$\begin{array}{cc} R\left(T\right) & =\frac{I_{1}\left(T\right)}{I_{2}\left(T\right)}=\frac{\varepsilon \left(T\right)\int_{\Delta \lambda_{1}}M_{b}\left(\lambda ,T\right)\cdot F_{1}\left(\lambda \right)d\lambda }{\varepsilon \left(T\right)\int_{\Delta \lambda_{2}}M_{b}\left(\lambda ,T\right)\cdot F_{2}\left(\lambda \right)d\lambda }\\ & =\frac{\int_{\Delta \lambda_{1}}M_{b}\left(\lambda ,T\right)\cdot F_{1}\left(\lambda \right)d\lambda }{\int_{\Delta \lambda_{2}}M_{b}\left(\lambda ,T\right)\cdot F_{2}\left(\lambda \right)d\lambda } \end{array}$$

This derivation demonstrates that, under the gray‐body approximation, the signal ratio *R*(*T*) is effectively decoupled from the target emissivity ε(λ, *T*), depending exclusively on the Planck function and the known system spectral responses, *F*_1_(λ) and *F*_2_(λ). Consequently, for a defined dual‐band optical architecture, a physically determined, monotonic mapping function *T* = *f*(*R*) exists. Once this relationship is established via experimental calibration or theoretical calculation, the target temperature *T* can be directly and uniquely retrieved from the measured ratio *R*, thereby effectively mitigating measurement errors arising from indeterminate emissivity within the tested range. Detailed physical principles of colorimetric thermometry are provided in Note S2.

To implement the proposed principle, a bifocal metalens was employed to exert independent and parallel phase control over the optical fields within the operating bands centered at λ_1_ = 9.760  ±  0.132 µm and λ_2_ = 10.561   ±   0.123 µm. These bands were selected within the LWIR atmospheric window (8–14 µm) to ensure high transmittance in practical scenarios. System calibration was conducted using a standard blackbody radiation source over a temperature range of $100^{\circ }C-450^{\circ }C$, where a series of dual‐band images were acquired under uniform thermal conditions. For each calibration temperature *T_i_*, the spatially averaged signal ratio *R_i_* = 〈*I*_1_〉/〈*I*_2_〉 was calculated over the full frame or a region of interest, and the resulting dataset (*R_i_*,*T_i_*) was subjected to curve fitting. Due to the intrinsic properties of the Planck function, this relationship exhibits excellent linearity or monotonic nonlinearity within finite temperature ranges, yielding a high‐precision calibration function *T* = *f*_cal_(*R*). This function implicitly accounts for all practical factors governing the system spectral responses *F*_1_(λ) and *F*_2_(λ). For target measurement, dual‐band images are captured and spatially registered; the local signal ratio *R*(*x*, *y*) = *I*_1_(*x*,*y*)/*I*_2_(*x*,*y*) is then computed for each pixel (*x*, *y*) and substituted into the calibration function *f*_cal_ to directly resolve the corresponding temperature*T*(*x*, *y*), ultimately generating a 2D thermal map that mitigates emissivity‐induced uncertainties.

It should be noted that while the gray‐body approximation (ε_1_ ≈ ε_2_) holds for a wide range of industrial materials, potential retrieval errors may arise when measuring selective radiators. If the target exhibits strong emission peaks or significant spectral gradients within the 9.6–10.6 µm window, the signal ratio *R*(*T*) would be modulated by the emissivity ratio $\frac{\varepsilon (\lambda_{1},T)}{\varepsilon (\lambda_{2},T)}$, leading to a deviation from the calibrated *f*(*T*). The selection of the 9.6 and 10.6 µm bands reflects a strategic trade‐off between measurement sensitivity and robustness. In ratiometric thermometry, a larger spectral separation (Δλ) enhances the temperature sensitivity *dR*/*dT*; however, it also increases the likelihood of deviating from the gray‐body. assumption (ε_1_/ε_2_ ≠ 1), thereby introducing significant systematic errors for non‐gray radiators. By constraining Δλ to approximately 1 µm, we ensure that the emissivity ratio for a wide range of materials remains near unity, maximizing the robustness of the retrieval model against unknown surface characteristics while maintaining high thermometric precision (relative error of approximately 3%). A more comprehensive analysis of the temperature retrieval errors arising from non‐gray body behavior is provided in Note S3.

Based on the theory of dual‐band ratio pyrometry governed by Planck's law, we designed a thermal imaging system targeting two infrared bands near 9.6 and 10.6 µm. In the experiments, due to the availability of commercial optical filters, the actual passbands were centered at 9.760 ± 0.132 µm and 10.561 ± 0.123 µm, respectively. Combined with a metalens capable of parallel manipulation of these two bands, the system was experimentally implemented. This approach mitigates emissivity‐induced uncertainty under the gray‐body assumption—a critical source of uncertainty—providing a robust theoretical and technical pathway for miniaturized, high‐precision non‐contact thermometry in complex environments. Future work may focus on optimizing band selection to enhance robustness against non‐gray body behavior, as well as developing advanced algorithms to compensate for environmental reflections and optical aberrations.

## 3 Design and Characterization

### 3.1 Design and Method

The metalens is designed to focus orthogonal linearly polarized light [13] (*x* and *y* polarizations) onto two spatially distinct focal points as shown in Figure 1a. Two focal points are laterally separated along the *x*‐axis and characterized by different focal lengths. To achieve this objective, the transmission phase profiles of the metalens were tailored for the two mutually orthogonal polarization states, expressed as follows:

(13) $$\begin{array}{c} \varphi_{X}\left(x,y\right)=-\frac{2\pi }{\lambda_{c}}\left[\sqrt{x^{2}+y^{2}+{d_{f_{X}}}^{2}+2xd_{f_{X}}\sin\theta }-d_{f_{X}}\right]\\ \varphi_{Y}\left(x,y\right)=-\frac{2\pi }{\lambda_{c}}\left[\sqrt{x^{2}+y^{2}+{d_{f_{Y}}}^{2}-2xd_{f_{Y}}\sin\theta }-d_{f_{Y}}\right] \end{array}$$

where φ_X_(*x*,*y*) and φ_Y_(*x*,*y*) denote the transmission phase profiles for x‐polarized and y‐polarized light, respectively. The proposed metalens is designed to achieve focusing over a broad spectral range of 9.6–10.6 µm. To ensure precise phase distribution at the target wavelength, the central wavelength λ_c_ in Equation 13 is set to 10.6 µm. The focal lengths are defined as $d_{f_{X}}=6.68\;\text{mm}$ and $d_{f_{Y}}=5.58\;\text{mm}$, with an off‐axis angle of θ = 10.4°.

**FIGURE 3:**
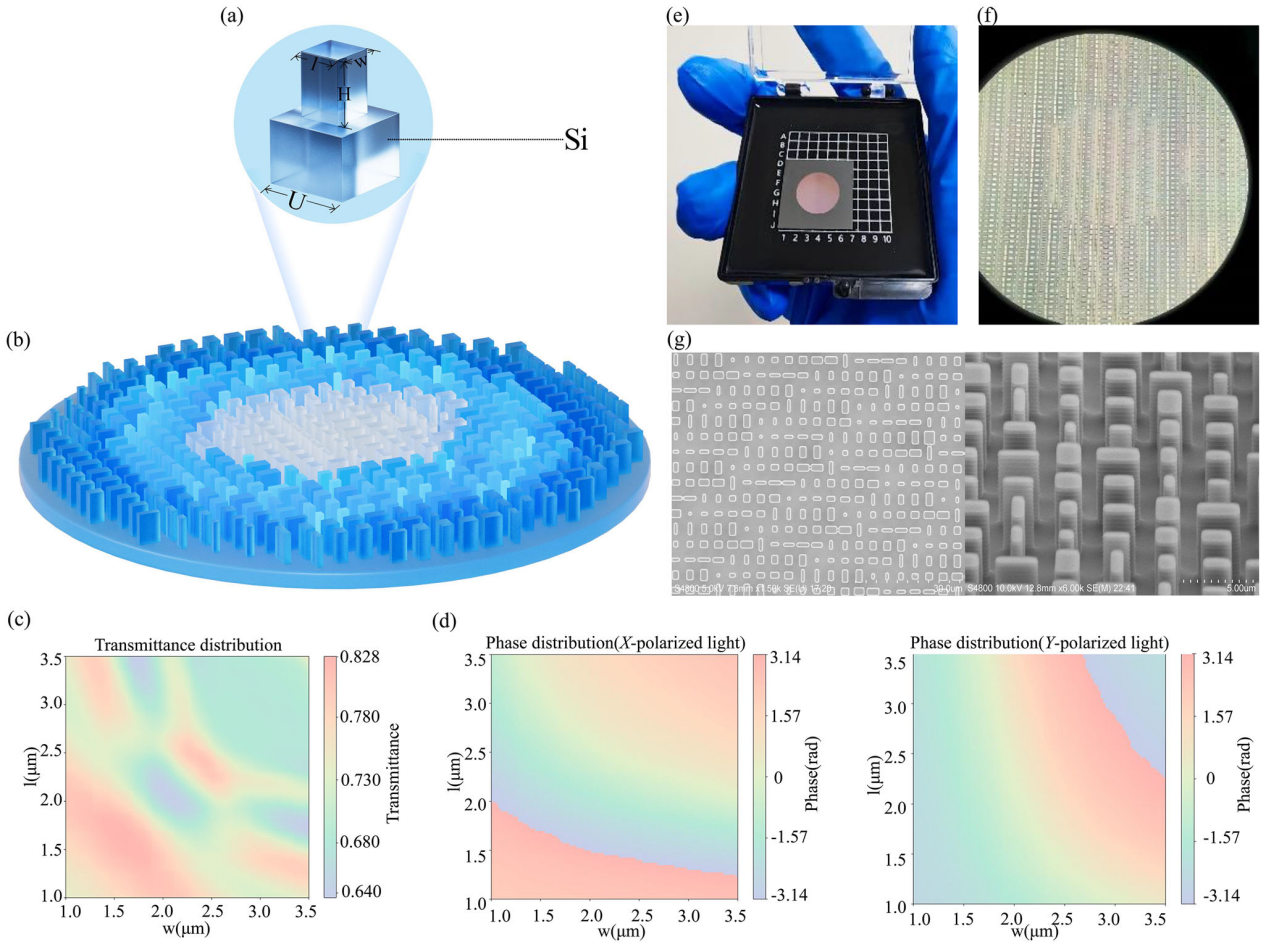
Design and characterization of the metalens unit cell. (a) Schematic illustration of the unit cell structure, consisting of an all‐silicon rectangular nanopillar. The pillar height is fixed at H=6 µm, and the unit cell is arranged in a square lattice with a periodicity of U=4 µm. (b) Perspective view of the metalens integrated on a silicon photonics platform. (c, d) Simulated transmittance (c) and transmission phase of *x*‐polarized and *y*‐polarized light (d) distributions varying with the silicon pillar length (*l*) and width (*w*). (e) Photograph of the fabricated device sample. (f) Optical micrograph displaying a partial view of the metalens structure. (g) Surface morphology revealed by scanning electron microscopy (SEM).

A bifocal metalens, as illustrated in Figure 3, was designed based on Equation (13). Figure 3a,b presents the structural design and characterization of the fundamental meta‐atom, which consists of an all‐silicon rectangular nanopillar. The pillar height is fixed at H=6 µm, with the units arranged in a square lattice with a period of U=4 µm (Figure 3a,b). To establish a precise mapping between geometric parameters and optical response, full‐wave numerical simulations were performed by sweeping the nanopillar length (*l*) and width (*w*) within the parameter space of 1–3.5 µm. The simulated transmittance (Figure 3c) and transmission phase (Figure 3d) distributions demonstrate that the design achieves full 0‐2π phase coverage while maintaining high transmission efficiency. This provides a comprehensive phase library for the independent wavefront manipulation of different polarization states.

### 3.2 Fabrication Process

The all‐silicon bifocal metalens designed in this work was fabricated on a silicon substrate using standard semiconductor nanofabrication protocols. First, a layer of hydrogen silsesquioxane (HSQ) electron‐beam resist was spin‐coated onto a cleaned, 500 µm ‐thick silicon substrate to serve as a hard mask for subsequent etching. The designed meta‐atom patterns were then defined directly into the resist using a high‐precision electron beam lithography (EBL) system. Following development to reveal the nanoscale HSQ mask, the patterns were precisely transferred into the underlying silicon substrate via reactive ion etching (RIE). By rigorously optimizing etching parameters—including gas flow ratios, RF power, and chamber pressure—we achieved high‐fidelity, anisotropic etching of the silicon, yielding the requisite high‐aspect‐ratio nanostructures. Finally, the residual HSQ mask was stripped using a buffered oxide etch (BOE) solution, resulting in the completed monolithic silicon metalens. The macroscopic appearance of the sample is shown in Figure 3e, while Figure 3f presents an optical micrograph capturing a representative region of the metalens.

### 3.3 Structural and Morphological Characterization

The structural characterization of the bifocal metalens fabricated in this study is shown in Figure 3e–g. To assess the fabrication quality and geometric fidelity of the nanostructures, detailed morphological characterization of the fabricated metalens was performed using scanning electron microscopy (SEM). As depicted in Figure 3g, the SEM micrographs clearly reveal the microscopic features of the metalens array and its constituent individual nanopillars (meta‐atoms).

The characterization results confirm the exceptional quality of the fabricated nanostructures. First, the meta‐atoms exhibit negligible surface roughness, indicating a robust reactive ion etching process that introduced no significant surface damage. Second, the nanopillars display excellent sidewall verticality, with measured sidewall angles exceeding $85^{\circ }$. This near‐ideal vertical profile is critical for achieving the designed optical phase modulation. Finally, the nanostructures demonstrate superior pattern fidelity, with dimensions, geometries, and periodicity showing excellent agreement with the initial design. This structural accuracy ensures a strong alignment between the experimental optical performance and theoretical simulations. Collectively, this high‐fidelity fabrication process lays a solid structural foundation for realizing the anticipated bifocal functionality of the metalens.

## 4 Experiment

In this study, we designed and fabricated a mid‐infrared broadband achromatic metalens with an effective operating bandwidth covering 9.6 to 10.6 µm. To achieve simultaneous non‐contact ranging and temperature measurement, we propose a scheme that integrates bifocal imaging with ratio pyrometry. From the broadband response of the metalens, two characteristic spectral bands—9.6 and 10.6 µm—were selected as information carriers. Specifically, the 10.6 µm band primarily encodes spatial defocus information, facilitating range retrieval via defocus analysis. Conversely, the relative radiant intensity ratio between the 9.6 and 10.6 µm bands constitutes the fundamental dataset for ratio pyrometry, enabling the retrieval of target temperature.

**FIGURE 4:**
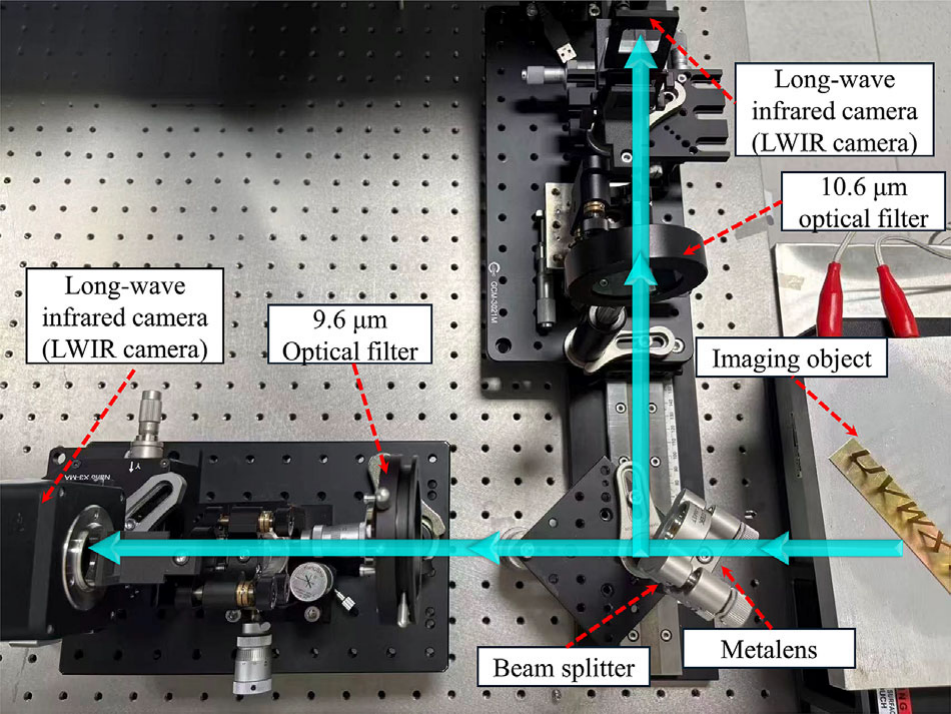
Schematic of the experimental setup for the bifocal metalens‐based system for simultaneous thermometry and ranging. The optical configuration is characterized on a pneumatic vibration‐isolated platform to maintain sub‐micron alignment stability. Thermal emission from the target object is modulated by the monolithic metalens and subsequently bifurcated into dual ratiometric channels (9.6 and 10.6 µm) via a beam splitter and narrow‐band filters. The resulting optical signatures are captured by synchronized LWIR cameras for simultaneous thermometry and depth retrieval.

The experimental system was constructed as shown in Figure 4. The fabricated broadband metalens served as the front‐end imaging element, precisely mounted at the optical entrance. To mitigate environmental stray radiation, a localized dark‐field environment was established by surrounding the optical path with light shields and black absorbing materials. To achieve spatial synchronization and precise separation of the dual‐band signals, a beam splitter was introduced into the imaging path, dividing the beam into two orthogonal detection channels. Each channel terminated with a high‐sensitivity infrared sensor equipped with a corresponding narrow‐bandpass filter—one centered at 9.6 µm and the other at 10.6 µm. The bandwidths of these filters were rigorously optimized to balance sufficient signal throughput with maximal suppression of spectral crosstalk between channels, satisfying the stringent independence and accuracy requirements of ratio pyrometry.

During the data acquisition process, a precision displacement stage was employed to translate the target object or the metalens along the optical axis, thereby introducing a sequence of known and controllable defocus amounts. At each prescribed defocus state, images from the dual infrared sensors were synchronously triggered and captured. This procedure facilitated the collection of multi‐defocus imaging data at the 10.6 µm band for depth‐from‐defocus (DFD) ranging, alongside corresponding 9.6 µm band data for dual‐band colorimetric thermometry. Consequently, a dataset comprised of paired and strictly registered dual‐band images was generated. Beyond encoding the spatial modulation information essential for distance retrieval, this dataset crucially provides the per‐pixel radiation intensity at both 9.6 and 10.6 µm, serving as the fundamental raw observations for subsequent colorimetric temperature calculations.

## 5 Results and Discussion

**FIGURE 5:**
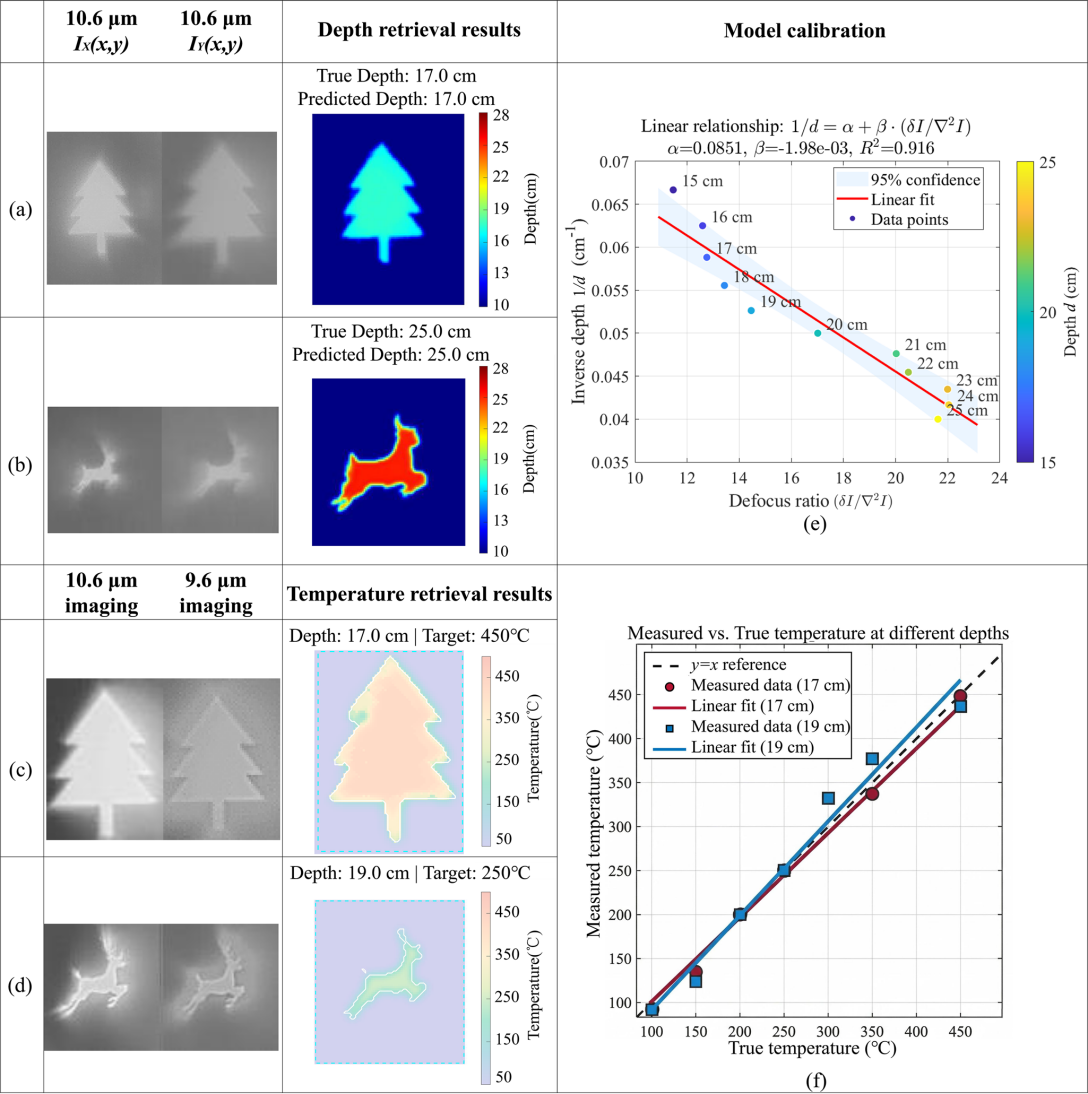
Experimental demonstration of simultaneous depth ranging and temperature measurement. (a,b) Captured defocused image pairs (*I*_X_ and *I*_Y_) at 10.6 µm and the corresponding reconstructed depth maps. The depth retrieval results show high accuracy with negligible error for targets placed at 17.0 and 25.0 cm. (c, d) Dual‐wavelength imaging results (9.6 and 10.6 µm) and the retrieved temperature maps for the ‘tree’ and ‘deer’ targets. The targets were heated to varying temperatures (100°C–450°C). (e) Linear calibration of the metalens depth sensor. (f) Quantitative assessment of temperature retrieval accuracy at depths of 17 and 19 cm via linear regression analysis.

Bifocal image pairs (*I*_X_,*I*_Y_) at 10.6 µm were experimentally acquired across a range of object distances (*d* ∈ [15, 25]cm) as shown in Figure 5a,b. To ensure the validity of the differential defocus theoretical model, the system was designed with a defocus difference of appropriate *d*_s_ value (corresponding to δσ in the theoretical framework in Equation 5). This minute defocus difference was selected to satisfy the stringent requirements for differential approximation; it approximates the variation in blur between the two images as a differential operation on the PSF width, while simultaneously maintaining an adequate signal‐to‐noise ratio.

During data processing, to quantify the mapping between the defocus signal and object depth, we acquired 11 calibration datasets (*n* = 11) across a working distance of 15.0–25.0 cm, the normalized defocus response, defined as *G* = δ*I*/∇^2^ *I*, was first extracted. Here, δ*I* = *I*_X_ − *I*_Y_ represents the intensity difference between the image pair, and ∇^2^ *I* denotes the Laplacian second‐order derivative of the averaged image. Subsequently, the system was linearly calibrated using the known inverse depth *P* = 1/*d*.

As shown in Figure 5e we performed a regression analysis of the true inverse depth (1/*d*, in cm^−1^) against the extracted normalized defocus response *G*. The scatter plot illustrates the relationship between the measured inverse depth (1/*d*) and normalized defocus response *G* for object distances ranging from 15.0–25.0 cm. The solid red line represents the linear regression fit with a coefficient of determination *R* ^2^ = 0.916 (*p* < 0.001), confirming the validity of the differential defocus model used for depth retrieval. Based on the fitted equation (1/*d* = α + β · (δ*I*/∇^2^ *I*)), the system exhibits a response slope of β ≈ −1.9773  ×  10^−3^ and an intercept of α ≈ 0.0851. This highly linear calibration curve demonstrates the system's ability to accurately retrieve target depth via measurement of the image defocus difference.

The high precision of this ranging model is fundamentally supported by the strategic selection of the 10.6 µm band as the primary depth information carrier. This wavelength capitalizes on the superior radiant intensity emitted by targets within the 100°C–450°C range, ensuring a high signal‐to‐noise ratio (SNR) during the extraction of the normalized defocus response. Such stability is critical for the robust execution of the Laplacian operator against ambient radiometric noise. Furthermore, although longer wavelengths inherently increase the diffraction‐limited PSF width, our monolithic metalens leverages precise phase modulation to maintain robust image contrast and sharp edge profiles at 10.6 µm. Consequently, the system achieves an ideal equilibrium between signal strength and spatial fidelity, effectively minimizing measurement deviations arising from both radiometric noise and diffraction limits.

Based on the proposed principle of colorimetric thermometry and the bifocal metalens imaging system, temperature calibration and measurement experiments were conducted at various object distances to validate the system's accuracy and stability in practical applications. In the experimental setup, the target was a copper mask coated with a uniform high‐emissivity layer. Its temperature was controlled by a high‐precision blackbody radiation source (accuracy ±0.5°C) within a range of 100°C–450°C, with temperature increments of 50°C. The target was positioned at six fixed distances from the metalens (15, 17, 19, 21, 23, and 25 cm) to ensure data acquisition under varying defocus conditions.

For each distance and temperature combination, the system synchronously acquired registered infrared images in the 9.6 µm and 10.6 µm bands as shown in Figure 5c,d. To mitigate the effects of noise and non‐uniformity, regional averaging was performed on each image, and the ratio of the integrated signal intensities of the two bands across the full field of view was calculated as follows:

(14) $$R\left(T,d\right)=\frac{\;\int \int \;I_{1}\left(x,y;T,d\right)\;dx\;dy}{\;\int \int \;I_{2}\left(x,y;T,d\right)\;dx\;dy}$$

where *d* denotes the object distance. Experimental results demonstrate that at each fixed object distance, the signal ratio *R* exhibits a highly consistent linear relationship with the true target temperature *T* (Figure 5f). Through least‐squares linear fitting, the calibration function was obtained:

(15) $$T_{\mathrm{fit}}=k_{d}\cdot R+b_{d}$$

Finally, the fitted linear relationships between measured and true temperatures at varying distances are presented in Figure 5f. It details the linear regression analyses at representative object distances of 17 and 19 cm, respectively. In these plots, markers denote experimental measurements, solid lines indicate the least‐squares fits, and dashed lines represent the ideal *y* = *x* reference. Quantitative analysis demonstrates a highly linear system response across the 100°C–450°C range: the coefficient of determination (*R* ^2^) is 0.9647 (RMSE = 22.24°C) at 17 cm, improving to 0.9787 (RMSE = 17.67°C) at 19 cm. These statistical metrics, all exceeding 0.95, confirm that the dual‐band response within this temperature interval aligns with gray‐body radiation theory predictions and that the system's optical response remains stable across the experimental distance range.

Furthermore, we analyzed the dependence of the temperature measurement results on the object distance. Although defocusing causes slight image blurring, the dual‐band signal ratio *R* is insensitive to defocus—the coefficient of variation for *R* values measured at the same temperature across different distances is less than 2%. This suggests that temperature retrieval based on the colorimetric method possesses excellent depth‐of‐field adaptability, eliminating the need for repetitive calibration at different distances.

Synthesizing the above results, this dual‐band metalens imaging system successfully achieved non‐contact, high‐precision temperature measurement of targets at 100°C–450°C within a range of 15–25 cm. This validates the feasibility of the temperature measurement method mitigating emissivity‐induced uncertainty based on the colorimetric principle in practical optical systems, laying an experimental foundation for the subsequent development of integrated, real‐time infrared thermal imaging systems. The observed measurement deviations primarily arise from fabrication‐induced phase distortions—such as sidewall tapering and surface roughness—which perturb the ideal point spread functions. Additionally, radiometric noise at lower thermal fluxes and systematic misalignment, coupled with the spatial sampling limits of the infrared sensor array, contribute to the remaining uncertainties in both temperature and depth retrieval. Beyond these instrumental artifacts, the intrinsic radiative properties of the target remain a critical constraint on measurement precision. For non‐gray radiators, a non‐unity ratio of spectral emissivities (ε_1_/ε_2_ ≠ 1) introduces a systematic gain error into the thermometric model, potentially distorting the ratiometric response. Prospective enhancements to the TRIM platform may involve the integration of multi‐spectral channels or adaptive compensation algorithms to more effectively decouple thermal signatures from complex, wavelength‐dependent emissivity profiles. A comprehensive analysis of these retrieval errors is detailed in Note S3.

While intrinsic target properties present fundamental measurement constraints, the operational robustness of the TRIM platform against external environmental perturbations is structurally guaranteed by its monolithic architecture and spectral engineering. By eschewing drift‐prone discrete configurations, the single‐aperture integration enforces intrinsic hardware‐level registration, effectively eradicating parallax and spatial mismatch. To safeguard radiometric fidelity, the bandwidths of the 9.6 µm and 10.6 µm filters were rigorously optimized to maximize signal throughput while suppressing spectral crosstalk. Furthermore, anchoring these operating bands within the long‐wave infrared (LWIR) atmospheric window deliberately bypasses the dominant absorption features of atmospheric H_2_O and CO_2_. This strategic spectral confinement ensures that the captured signatures are overwhelmingly driven by the target's intrinsic emission rather than ambient background noise. Collectively, these synergistic mechanisms establish a resilient physical framework for high‐precision, multidimensional perception in complex real‐world scenarios.

As systematically benchmarked in Table 1, we compare our approach with representative metalens‐based sensing studies. Prior work has achieved notable progress across distinct sensing modalities. For example, Zhao et al. employed polarization multiplexing to mitigate scattering and enhance contrast in turbid underwater imaging, while Guo et al., inspired by the jumping spider, demonstrated compact and efficient single‐shot passive depth sensing [27]. In addition, Luo et al. addressed accurate thermography of objects with unknown emissivity using a dual‐band metalens combined with colorimetric methods. Despite these advances, existing approaches remain largely confined to “discrete” functionalities, focusing on either ranging or thermometry, and are unable to simultaneously acquire thermal and geometric information using a single optical element. Here, we bridge this gap by demonstrating a physical‐level fusion of high‐precision radiometric thermometry (relative error ∼3%) and passive ranging (within a 95% confidence interval) on a monolithic dual‐focus metalens. The proposed TRIM platform represents a strategic step toward unifying geometric and thermodynamic sensing within a single aperture.

**TABLE 1:** Performance comparison with representative metalens sensing systems.

Work / Reference	Metalens diameter (D)	Operating band	Measurement distance range	Measurement temperature range	Accuracy (temp / ranging)	Primary functions
Zhao et al. (2021) [30]	3 mm	Near‐infrared (800 nm)	6 cm and 15 cm (experimental demonstration)	Not applicable (focus on underwater polarization imaging)	Ranging: not quantitatively specified (focus on image contrast/EME enhancement of ∼10–12x)	Polarization‐enhanced imaging and depth estimation
Guo et al. (2019) [27]	3 mm	Visible (532 nm)	30–40 cm (high confidence range)	Not applicable (focus on passive depth sensing)	Ranging: relative error approx. 5% (within the 30–40 cm range)	Depth sensing and imaging
Luo et al. (2024) [21]	2 cm	LWIR (9.5/12.5 µm)	Not applicable (focus on colorimetric thermography)	60°C–180°C	Thermography: error reduced by 50.16% (compared to commercial cameras without preset emissivity)	Colorimetric thermography and imaging
Our work	12 mm	LWIR (9.6 / 10.6 µm)	15–25 cm	100°C – 450°C	Thermography: mean absolute error 13.58°C (relative error ∼3%) Ranging: deviations within 95% confidence interval (R^2^ = 0.916)	Thermometry, ranging, and imaging

While metasurface‐based sensors have achieved impressive performance in individual modalities—from bio‐inspired depth reconstruction to thermography that mitigates emissivity‐induced uncertainty—the synchronous acquisition of physical and spatial information remains a major challenge. Our architecture enables pixel‐level spatiotemporal synchronization through monolithic integration, thereby eliminating parallax and registration errors inherent to discrete multi‐sensor systems. Importantly, the integrated shared‐path design further enables a distance‐informed thermal calibration mechanism: by incorporating real‐time distance into the radiometric model, the system dynamically compensates for atmospheric attenuation and path‐dependent radiative losses. This synergy is expected to deliver improved thermal fidelity in complex 3D environments, outperforming conventional disjointed systems that rely on static or estimated distance assumptions. By overcoming the limitations of traditional discrete architectures in synchronous multidimensional acquisition without increasing system complexity, our work provides a robust and integrated solution for next‐generation chip‐scale intelligent perception platforms.

To fully realize the potential of chip‐scale platforms in macroscopic, real‐world environments, future development must address both geometric scalability and environmental heterogeneity. While the demonstrated 15–25 cm sensing range provides a robust proof of concept for the monolithic TRIM architecture, extending this range requires deliberate optical scaling. Within the depth‐from‐defocus framework, sensitivity to defocus inherently diminishes at longer distances due to increased depth of field. Maintaining high‐fidelity depth retrieval at extended ranges therefore necessitates compensating for this attenuation by scaling the effective aperture or precisely engineering the phase‐induced focal shift.

In addition, practical deployment involves non‐uniform scenes with complex thermal gradients and heterogeneous materials. The TRIM platform mitigates these challenges through the intrinsic pixel‐level independence of its ratiometric thermometry. By extracting the dual‐band signal ratio on a per‐pixel basis, the system effectively decouples temperature retrieval from local variations in absolute emissivity. Combined with the use of LWIR atmospheric window to minimize ambient absorption, these attributes establish the monolithic metalens as a scalable and robust architecture for next‐generation multidimensional perception.

## 6 Conclusion

This study successfully constructed a 12‐mm aperture infrared fusion perception system based on a bifocal metalens, which enables simultaneous distance and temperature sensing in the long‐wave infrared (LWIR) band (9.6 and 10.6 µm) through optical‐level integration and physical‐level synergy for the first time. Obviating the need for complex beam‐splitting architectures or multi‐sensor stacking, our system exploits the wavelength selectivity and focusing flexibility of diffractive optics. By generating dual‐band image pairs with a fixed focal shift in a single optical system, we realize multidimensional information acquisition within a monolithic, single‐aperture optical framework.

In the domain of distance sensing, experimental results confirm a significant linear correlation between the normalized defocus response and inverse depth. Under defined optical constraints, this stable mapping provides a robust physical basis for Depth from Defocus (DfD) calculations, validating the feasibility of passive ranging utilizing the dispersive properties of the metalens. Regarding temperature sensing, the system demonstrates superior performance in ratio thermometry. Across a broad temperature range of 100°C–450°C, the dual‐band radiation intensity ratio exhibits a strong linear correlation with the target temperature (*R* ^2^ > 0.95). Crucially, this ratio‐based approach effectively mitigates the uncertainties associated with target surface emissivity—a limitation inherent to single‐band methods—thereby significantly enhancing the robustness of non‐contact thermometry.

In summary, by harnessing the subwavelength structural engineering of metalens, we have successfully integrated dual‐band imaging, bifocal control, and diffraction‐limited focusing into a single optical system. This work establishes a critical technological pathway for the miniaturization and integration of infrared perception systems. Future efforts will focus on inverse design optimization to improve optical efficiency, coupled with multi‐physics simulations to enhance environmental adaptability. Furthermore, the incorporation of deep learning algorithms promises to further elevate calculation precision in complex scenarios, advancing infrared sensing technology toward greater integration and intelligence.

## Author Contributions

Man Yuan carried out the experimental design, literature search, and the execution of the experiments. Yuqing Zhang and Xiaoyun He carried out the implementation of the experiments. Wangzhe Zhou contributed to data curation and visualization. Xinpeng Jiang and Yiyi Li were involved in formal analysis and software support. Xin He provided administrative and technical support. Zhaojian Zhang, Jiagui Wu, Yuanmu Yang, and Junbo Yang performed the supervision, provided resources, and critically revised the manuscript. Zhaojian Zhang reviewed and edited the manuscript. Jiagui Wu and Junbo Yang were responsible for funding acquisition and project administration.

## Funding

This work was supported by the National Key Research and Development Program of China (2022YFF0706005), the National Natural Science Foundation of China (62275271, 62305387, 12272407, 62405037, 62275269), and the Chongqing Natural Science Foundation (CSTB2024NSCQ‐MSX0581, CSTB2024NSCQ‐LZX0033).

## Conflicts of Interest

The authors declare no conflicts of interest.

## Supporting information




**Supporting File**: advs75776‐sup‐0001‐SuppMat.docx.

## Data Availability

The data that support the findings of this study are available on request from the corresponding author. The data are not publicly available due to privacy or ethical restrictions.

## References

[1] A. Wirth‐Singh , J. E. Fröch , Z. Han , et al., “Large Field‐of‐View Thermal Imaging via All‐Silicon Meta‐Optics,” Applied Optics 62, no. 20 (2023): 5467–5474, 10.1364/AO.493555. 37706864

[2] L. Huang , Z. Han , A. Wirth‐Singh , et al., “Broadband Thermal Imaging Using Meta‐Optics,” Nature Communications 15, no. 1 (2024): 1662, 10.1038/s41467-024-45904-w. PMC10891089 38395983

[3] C. Zhao , Z. Liu , and W. Huang , “Wide Field‐Of‐View Metalens Array for the Long‐Wavelength Infrared,” in Conference on Infrared, Millimeter, Terahertz Waves and Applications (IMT2022) (SPIE, 2023), 746–749.

[4] N. Zhang , Q. Li , J. Chen , et al., “Design of an All‐Dielectric Long‐Wave Infrared Wide‐Angle Metalens,” Chinese Physics B 31, no. 7 (2022): 074212, 10.1088/1674-1056/ac4026.

[5] S. Yue , Y. Liu , R. Wang , et al., “All‐Silicon Polarization‐Independent Broadband Achromatic Metalens Designed for the Mid‐Wave and Long‐Wave Infrared,” Optics Express 31, no. 26 (2023): 44340–44352, 10.1364/OE.506471. 38178507

[6] F. L. Meng , J. L. Liu , J. X. Yue , et al., “Design of a Polarization‐Insensitive Broadband Achromatic Metalens for Mid‐Wave Infrared Detector,” Optics Letters 49, no. 19 (2024): 5563–5566, 10.1364/OL.541487. 39353007

[7] X. Li , S. Chen , D. Wang , X. Shi , and Z. Fan , “Transmissive Mid‐Infrared Achromatic Bifocal Metalens With Polarization Sensitivity,” Optics Express 29, no. 11 (2021): 17173–17182, 10.1364/OE.424887. 34154265

[8] X. Liu , G. Zhang , Y. Huang , Y. Wang , and F. Qi , “Two‐Dimensional Temperature and Carbon Dioxide Concentration Profiles in Atmospheric Laminar Diffusion Flames Measured by Mid‐Infrared Direct Absorption Spectroscopy at 4.2 µm,” Applied Physics B 124, no. 4 (2018): 61, 10.1007/s00340-018-6930-0.

[9] R. Estevâm da Silva , “Far‐Infrared Slab Lensing and Subwavelength Imaging in Crystal Quartz,” Physical Review B 86, no. 15 (2012): 155152, 10.1103/PhysRevB.86.155152.

[10] Q. Guo , E. Alexander , and T. Zickler , “Focal Track: Depth and Accommodation With Oscillating Lens Deformation,” in 2017 IEEE International Conference on Computer Vision (ICCV) (2017), 966–974, 10.1109/ICCV.2017.110.

[11] M. Khorasaninejad , W. T. Chen , A. Y. Zhu , et al., “Multispectral Chiral Imaging With a Metalens,” Nano Letters 16, no. 7 (2016): 4595–4600, 10.1021/acs.nanolett.6b01897. 27267137

[12] M. Y. Shalaginov , S. An , Y. Zhang , et al., “Reconfigurable All‐Dielectric Metalens With Diffraction‐Limited Performance,” Nature Communications 12, no. 1 (2021): 1225, 10.1038/s41467-021-21440-9. PMC7900249 33619270

[13] J. Ma , T. Kang , Z. Ke , et al., “Tunable Far‐Infrared Polarization Imaging Based on VO_2_ Metasurfaces,” Advanced Optical Materials 12, no. 11 (2024): 2302390, 10.1002/adom.202302390.

[14] R. Li , J. Wei , L. Wang , Y. Ma , and Y. Li , “Restoration of Infrared Metalens Images With Deep Learning,” Optics Communications 552 (2024): 130069, 10.1016/j.optcom.2023.130069.

[15] Y.‐C. Chen , W.‐L. Hsu , Q.‐C. Zeng , et al., “Broadband Achromatic Thermal Metalens With a Wide Field of View Based on Wafer‐Level Monolithic Processes,” Applied Physics Letters 125, no. 5 (2024): 051702, 10.1063/5.0220043.

[16] K. Huang , Y. Yang , R. Tang , et al., “Review of the Development of Lunar Laser Ranging,” Astronomical Techniques and Instruments 1, no. 6 (2024): 295–306, 10.61977/ati2024048.

[17] J. Sun and W. Fan , “Error Analysis and Accurate Temperature Measurement Method of Infrared Thermal Imaging Long‐Distance Temperature Measurement in Underground Mine,” Meitan Xuebao/Journal of the China Coal Society 47, no. Compendex (2022): 1709–1722, 10.13225/j.cnki.jccs.2021.1830.

[18] W. Wu , G. Tang , C. Liu , et al., “XingHuan Visible and Uncooled Multispectral Infrared Camera for Wildfire Detection: Algorithm Description and Initial Validation,” IEEE Journal of Selected Topics in Applied Earth Observations and Remote Sensing 18 (2025): 20149–20162, 10.1109/JSTARS.2025.3592843.

[19] T. Kruczek , “Conditions for Use of Long‐Wave Infrared Camera to Measure the Temperature of the Sky,” Energy 283 (2023): 128466, 10.1016/j.energy.2023.128466.

[20] H. Runheng and K.‐N. Liou , “Remote Sounding of the Cirrus Optical Depth and Temperature From 3.7 and 11 Micrometer Windows,” Advances in Atmospheric Sciences 1, no. 2 (1984): 150–164, 10.1007/BF02678128.

[21] Z. Luo , P. Zhang , H. Hou , et al., “Colorimetric Thermography by a Long‐Infrared Dual‐Band Metalens,” Advanced Science 12, no. 2 (2025): 2408683, 10.1002/advs.202408683. 39560152 PMC11727133

[22] C.‐Y. Lin and W.‐S. Yao , “Compensation for Vanadium Oxide Temperature With Stereo Vision on Long‐Wave Infrared Light Measurement,” Sensors 22, no. 21 (2022): 8302, 10.3390/s22218302. 36365999 PMC9655447

[23] Á. S. Machado , M. Cañada‐Soriano , I. Jimenez‐Perez , et al., “Distance and Camera Features Measurements Affect the Detection of Temperature Asymmetries Using Infrared Thermography,” Quantitative InfraRed Thermography Journal 21, no. 2 (2024): 69–81, 10.1080/17686733.2022.2143227.

[24] S. Weng , D. Hou , Z. Yuan , J. Ding , and P. Liang , “Infrared Temperature Measurement of Slender Irregular Tubes by Thermal Image Guided Scanning,” Measurement Science and Technology 36, no. 5 (2025): 055009, 10.1088/1361-6501/adccec.

[25] Y. Wu , F. Wang , T. Deng , J. Zhang , G. Xia , and Z. Wu , “Long‐Range Temperature Sensing Based on Forward Brillouin Scattering in Highly Nonlinear Fiber,” Optics & Laser Technology 181 (2025): 111619, 10.1016/j.optlastec.2024.111619.

[26] H. K. M. Tanaka , “Muopause Sounder to Derive Vertically Averaged Atmospheric Temperature Underneath the Muopause With the Distance of Flight Muography Technique,” Scientific Reports 15, no. 1 (2025): 25938, 10.1038/s41598-025-09090-z. 40676020 PMC12271455

[27] Q. Guo , Z. Shi , Y.‐W. Huang , et al., “Compact Single‐Shot Metalens Depth Sensors Inspired by Eyes of Jumping Spiders,” Proceedings of the National Academy of Sciences 116, no. 46 (2019): 22959–22965, 10.1073/pnas.1912154116. PMC6859311 31659026

[28] L. Wang , Z. Hu , W. Shui , and F. Wu , “Pressure and Distance Measurements Under Temperature Interference by Using Impedance Change of Spiral Conductive Polymer Composite,” Measurement 241 (2025): 115696, 10.1016/j.measurement.2024.115696.

[29] I. Kim , R. J. Martins , J. Jang , et al., “Nanophotonics for Light Detection and Ranging Technology,” Nature Nanotechnology 16, no. 5 (2021): 508–524, 10.1038/s41565-021-00895-3. 33958762

[30] F. Zhao , R. Lu , X. Chen , et al., “Metalens‐Assisted System for Underwater Imaging,” Laser & Photonics Reviews 15, no. 8 (2021): 2100097, 10.1002/lpor.202100097.

[31] S. Li , W. Zhou , Y. Li , et al., “Collision of High‐Resolution Wide FOV Metalens Cameras and Vision Tasks,” Nanophotonics 14, no. 3 (2025): 315–326, 10.1515/nanoph-2024-0547. 39967773 PMC11831395

[32] G. Ortolano and I. Ruo‐Berchera , “Quantum Target Ranging for LiDAR,” Physical Review Research 7, no. 2 (2025): L022059, 10.1103/PhysRevResearch.7.L022059.

[33] X. Jiang , J. Nong , X. Li , et al., “Laser‐Adaptive Inverse‐Design Metamaterials for Durable Regulation From Visible‐Infrared‐LiDAR Compatible Camouflage to Optical Limiter,” Laser & Photonics Reviews 19, no. 22 (2025): 00881, 10.1002/lpor.202500881.

[34] S. Wang , P. C. Wu , V.‐C. Su , et al., “A Broadband Achromatic Metalens in the Visible,” Nature Nanotechnology 13, no. 3 (2018): 227–232, 10.1038/s41565-017-0052-4. 29379204

[35] H. Li , K. Luo , D. P. Tsai , et al., “Broadband, Wide‐Angle, and Versatile Metasurface Illusions With Inverse Synthetic Aperture Radar Imaging,” Advanced Science 12, no. 18 (2025): 2416172, 10.1002/advs.202416172. 39921457 PMC12079480

[36] C. Feng , T. He , Y. Shi , et al., “Diatomic Metasurface for Efficient Six‐Channel Modulation of Jones Matrix (Laser Photonics Rev. 17(8)/2023),” Laser & Photonics Reviews 17, no. 8 (2023): 2370040, 10.1002/lpor.202370040.

[37] C. Feng , Q. Zhong , T. He , et al., “Bilayer Triatomic Metasurface‐Driven Minimalist Full‐Channel Modulation of Jones Matrix,” Laser & Photonics Reviews 20, no. 4 (2026): 01590, 10.1002/lpor.202501590.

[38] W. Yang , S. Xiao , Q. Song , et al., “All‐Dielectric Metasurface for High‐Performance Structural Color,” Nature Communications 11, no. 1 (2020): 1864, 10.1038/s41467-020-15773-0. PMC7171068 32313078

[39] Z. Wei , W. Xu , S. Dong , et al., “Ultraprecision, High‐Capacity, and Wide‐Gamut Structural Colors Enabled by a Mixture Probability Sampling Network,” Light: Science & Applications 15, no. 1 (2026): 164, 10.1038/s41377-025-02122-3. PMC12976068 41807352

[40] D. Xue , X. Dun , Z. Wei , et al., “Collimated Flat‐Top Beam Shaper Metasurface Doublet Based on the Complex‐Amplitude Constraint Gerchberg–Saxton Algorithm,” Nanophotonics 13, no. 8 (2024): 1379–1385, 10.1515/nanoph-2023-0719. 39679233 PMC11636518

[41] W. Xu , L. Hu , K. Shao , et al., “Design of Arbitrary Energy Distribution Beam Splitters Base on Multilayer Metagratings by a Hybrid Evolutionary Particle Swarm Optimization,” Optics Express 31, no. 25 (2023): 41339–41350, 10.1364/OE.502125. 38087535

[42] M. Khorasaninejad , W. T. Chen , R. C. Devlin , J. Oh , A. Y. Zhu , and F. Capasso , “Metalenses at Visible Wavelengths: Diffraction‐Limited Focusing and Subwavelength Resolution Imaging,” Science 352, no. 6290 (2016): 1190–1194, 10.1126/science.aaf6644. 27257251

[43] Y. Nagase , T. Kushida , K. Tanaka , T. Funatomi , and Y. Mukaigawa , “Shape From Thermal Radiation: Passive Ranging Using Multi‐Spectral LWIR Measurements,” in 2022 IEEE/CVF Conference on Computer Vision and Pattern Recognition (CVPR) (IEEE, 2022), 12651–12661, 10.1109/CVPR52688.2022.01233.

[44] G. Liu , J. Li , J. Li , S. Yue , and R. Zhou , “Estimation of Nighttime Aerosol Optical Depths Using Atmospheric Infrared Sounder Longwave Radiances,” Geophysical Research Letters 51, no. 8 (2024): 2023GL108120, 10.1029/2023GL108120.

[45] X. Li , L. Li , Q. Huang , and P. Wang , “Influence of Thermocouple Angles and Wire Distance on Temperature Measurement,” Case Studies in Thermal Engineering 49 (2023): 103221, 10.1016/j.csite.2023.103221.

[46] S. Chaudhuri and A. N. Rajagopalan , Depth from Defocus: A Real Aperture Imaging Approach (Springer, 1999), 10.1007/978-1-4612-1490-8.

