# Supplementary material for: TRIM: Simultaneous Thermometry, Ranging, and Imaging via a Monolithic Metalens
Source: Adv Sci (Weinh). 2026 May 28:e75776. Online ahead of print. doi: 10.1002/advs.75776 (PMC13336119; doi:10.1002/advs.75776)
Supplement: Supplementary file 1 — Supporting File: advs75776‐sup‐0001‐SuppMat.docx. [file ADVS-9999-e75776-s001.docx]

Supporting Information

**TRIM: Simultaneous Thermometry, Ranging, and Imaging via A Monolithic Metalens**

*Man Yuan^1^, Yuqing Zhang^2^, Wangzhe Zhou^1^, Zhaojian Zhang^1*^, Xiaoyun He^2^, Xinpeng Jiang^1^, Yiyi Li^1^, Xin He^1^, Jiagui Wu^2*^, Yuanmu Yang^3*^, Junbo Yang^1*^*

*^1^College of Science, National University of Defense Technology, Changsha 410073, China*

*^2^ School of Physical Science and Technology, Southwest University, Chongqing 400715, China*

*^3^* *State Key Laboratory of Precision Measurement Technology and Instruments, Department of Precision Instrument Tsinghua University*

**Corresponding author: yangjunbo@nudt.edu.cn*

mgh@swu.edu.cn

ymyang@tsinghua.edu.cn

zhangzhaojian@nudt.edu.cn

**Supplementary Note 1. Quantification of truncation error and validity of the first-order approximation**

First, we establish the fundamental relationship between the radius of the circle of confusion,$\sigma$, and the object distance,$d$, based on the thin-lens equation:

$\sigma(d,f)=\left[\left(\frac{1}{f}-\frac{1}{d}\right)d_{s}\right]K$ (S1)

where $d_{s}$ denotes the image distance and $K$ is the aperture radius.

The variation in image intensity $I$ as a function of the blur parameter $\sigma^{2}$ is governed by the diffusion equation:

$\frac{\partial I}{\partial(\sigma^{2})}=\frac{1}{2}\nabla^{2}I$ (S2)

To rigorously evaluate the validity of the differential refocusing approximation under our specific design parameters (focal lengths $f_{X}=6.68$mm and $f_{Y}=5.58$mm), we quantify the truncation error by expanding the depth retrieval model to include higher-order terms. The image intensity difference $\delta I=I_{X}-I_{Y}$ induced by a minute variation in focal length can be expressed as a Taylor series expansion with respect to the variance of the circle of confusion ($\Delta\sigma^{2}$).

(S3)

By substituting the spatial derivative relationships, the above equation can be rewritten as follows:

$\delta I\approx\frac{1}{2}(\nabla^{2}I)\Delta(\sigma^{2})+\frac{1}{8}(\nabla^{4}I)[\Delta(\sigma^{2})]^{2}$ (S4)

Here, the first term on the right side of the equation is used in our first-order depth retrieval model (as detailed in Equation (6) of the main text). The second term represents the primary truncation error of the system, where is the bilaplacian operator, corresponding to higher-order image curvature.

Consequently, the foundational first-order and refined higher-order depth measurement formulations are given by:

$\begin{matrix} \text{1st-order: }\frac{1}{d}=\alpha+\beta\left(\frac{\delta I}{\nabla^{2}I}\right) \\ \text{Higher-order: }\frac{1}{d}\approx\alpha\text{+}\beta\left(\frac{\delta I\text{-}\frac{1}{8}\nabla^{4}I[\Delta(\sigma^{2})]^{2}}{\nabla^{2}I}\right) \end{matrix}$ (S5)

Despite a physical difference of approximately 1.1 mm between the designed focal lengths of the two channels, the first-order approximation remains valid within the tested measurement range in our system. Fundamentally, the validity of the Taylor expansion relies on the variance of the circle of confusion mapped onto the sensor plane ($\Delta\sigma^{2}$), rather than the absolute magnitude of the focal length difference. Given the operational constraints of working distance, image distance, and effective aperture, the variation induced by the focal length difference remains a sufficiently small perturbation, thereby guaranteeing the rapid convergence of the series expansion. Furthermore, the magnitude of the truncation error is not solely determined by $\Delta\sigma^{2}$; it is also primarily constrained by the image bilaplacian, $\nabla^{4}I$ (the fourth-order spatial derivative). Because the spatial power spectrum of natural scenes typically exhibits a sharp roll-off, this higher-order spatial derivative approaches a minimal value for the low- and mid-frequency structures that dominate natural images. Consequently, quantitative analysis demonstrates that the truncation error yields non-trivial values primarily in highly localized sub-pixel regions containing extreme high-frequency features, such as singular edges or pure high-frequency noise.

Figure S1 shows the comparison of linear regression for depth estimation between the first-order approximation and the higher-order correction model. It is shown that across a measurement range of 15 cm to 25 cm, the fitted curves and 95% confidence intervals of the 1st-order approximation model and the higher-order correction model exhibit remarkable congruence (with coefficients of determination $R^{2}$ exceeding 0.915 for both). This visually demonstrates that the introduction of higher-order terms does not significantly alter the linear mapping between inverse depth and the defocus ratio, further validating the adequacy and precision of the current first-order approximation for practical applications.

In summary, the first-order approximation is rigorous and physically self-consistent within the effective measurement range of this bifocal metalens system. The impact of higher-order truncation errors on the ultimate depth retrieval accuracy is effectively contained to a negligible level.





**Figure S1 | Comparison of linear regression for depth estimation between the first-order approximation and the higher-order correction model.** The plot illustrates the mapping relationship between the target inverse depth ($1/d$) and the defocus ratio ($\delta I/\nabla^{2}I$) over a testing range of 15 cm to 25 cm. The solid blue line and solid dots represent the results of the first-order approximation ($R^{2}=0.9159$), while the dashed red line and hollow rhombuses depict the results after incorporating the higher-order truncation error correction ($R^{2}=0.9153$). Shaded regions denote the 95% confidence intervals for both datasets. This visually confirms that the impact of higher-order truncation error on the overall depth estimation is negligible, validating the efficacy of the first-order approximation model.

To quantify the performance of the first-order approximation in spatial localization, we performed depth reconstruction tests from 15 cm to 25 cm. As shown in the depth error analysis plot (Figure S2), the predicted depths closely align with the ideal reference line. The statistical deviation confirms that the truncation error remains negligible, as predicted by the theoretical derivation.


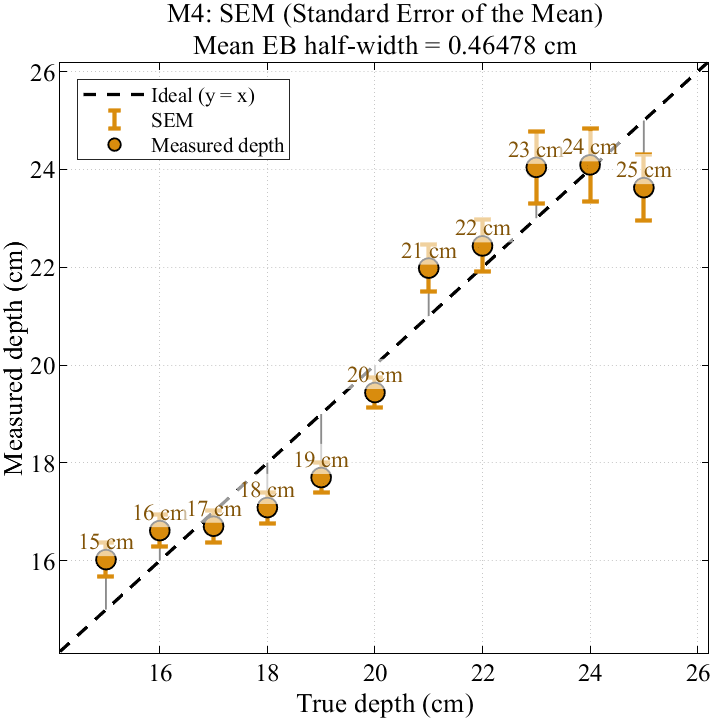


**Figure S2. Depth estimation error bar analysis.** This plot illustrates the correspondence between the reconstructed depth and the true depth over a testing range of 15 cm to 25 cm. Orange circles represent the mean measured depth, and the error bars denote the Standard Error of the Mean (SEM), with a mean half-width of 0.46478 cm. The high degree of congruence between the experimental data and the black dashed line ($y=x$) validates the rigor and reliability of the first-order approximation model for natural scenes.

**Supplementary Note 2. Principle of temperature measurement based on Planck's law**

The spectral radiant exitance, $M(\lambda,T)$, of electromagnetic radiation emitted by a blackbody is described by Planck's law as:

$M_{b}(\lambda,T)=\frac{2\pi hc^{2}}{\lambda^{5}}\cdot\frac{1}{\exp\left(\frac{hc}{\lambda kT}\right)-1}$ （S6）

Where $M_{_{b}}(\lambda,T)$ denotes the spectral radiant exitance ($W\cdot m^{-3}$), $T$ represents the absolute temperature (K), and $\lambda$ is the wavelength (fixed at $10 .6\mu\text{m}$ in this study). The physical constants include Planck’s constant $h$ ($6.626\times10^{-34}$J·s), the speed of light $c$ ($3.0\times10^{8}$m/s), and the Boltzmann constant $k$($1.38\times10^{-23}$J/K).

Real objects are typically modeled as gray bodies, with their radiative properties corrected by the emissivity, $\varepsilon$:

$M_{\text{actual}}(\lambda,T)=\varepsilon(\lambda,T)\cdot M_{\text{b}}(\lambda,T)$ (S7)

Real objects are typically modeled as gray bodies, with their radiative properties corrected by the emissivity,$\varepsilon$. This emissivity, $\varepsilon(\lambda,T)$, is a function of surface characteristics, wavelength, temperature, and viewing angle, representing the most pivotal and complex parameter in infrared thermometry. Consequently, the radiant intensity detected by the sensor is obtained through integration:

$M\left(T\right)=\int M\left(\lambda,T\right)d\lambda=\int_{\lambda_{\text{start}}}^{\lambda_{\text{end}}} M_{\text{b}}\left(\lambda,T\right)*\varepsilon\left(\lambda,T\right)*F\left(\lambda\right)d\lambda\text{ }$ (S8)

Here, $F\left(\lambda\right)$ denotes the system transmittance, which accounts for the spectral response of the infrared sensor, the spectral transmittance of the metal, and the atmospheric window. The range $\lambda_{\text{start}}\approx\lambda_{\text{end}}$ defines the operating bandwidth of the sensor, which is $8\text{-}14 \mu\text{m}$ for the long-wave infrared (LWIR) sensor used in this study.

In practical measurements, the detector captures the integrated radiant power within a finite optical bandwidth,$\Delta\lambda$. For a dual-channel system with center wavelengths $\lambda_{1}$ and $\lambda_{2}$, and corresponding bandwidths $\Delta\lambda_{1}$ and$\Delta\lambda_{2}$, the output signals $I_{1}(T)$ and $I_{2}(T)$are expressed as:

$\begin{matrix} I_{1}(T)=\int_{\Delta\lambda_{1}} \varepsilon(\lambda,T)\cdot M_{b}(\lambda,T)\cdot F_{1}(\lambda) d\lambda\\ I_{2}(T)=\int_{\Delta\lambda_{2}} \varepsilon(\lambda,T)\cdot M_{b}(\lambda,T)\cdot F_{2}(\lambda) d\lambda\end{matrix}$ (S9)

where $F_{1}(\lambda)$ and $F_{2}(\lambda)$ represent the system spectral response functions for the two respective channels. The fundamental principle of colorimetric thermometry relies on the ratio of these two signal bands, $R(T)$, to derive the target temperature:

$R(T)=\frac{I_{1}(T)}{I_{2}(T)}=\frac{\int_{\Delta\lambda_{1}} \varepsilon(\lambda,T)\cdot M_{b}(\lambda,T)\cdot F_{1}(\lambda) d\lambda}{\int_{\Delta\lambda_{2}} \varepsilon(\lambda,T)\cdot M_{b}(\lambda,T)\cdot F_{2}(\lambda) d\lambda}$ (S10)

If the target can be approximated as a gray body, implying that its emissivity exhibits negligible spectral dependence, $\varepsilon$ can be treated as a temperature-dependent constant, $\varepsilon(T)$, within the specified narrow bands $\Delta\lambda_{1}$ and$\Delta\lambda_{2}$. Under this assumption, the above equation simplifies to^[21]^:

$R\left(T\right)=\frac{\varepsilon\left(T\right)\int_{\Delta\lambda_{1}} M_{b}\left(\lambda,T\right)\cdot F_{1}\left(\lambda\right)d\lambda}{\varepsilon\left(T\right)\int_{\Delta\lambda_{2}} M_{b}\left(\lambda,T\right)\cdot F_{2}\left(\lambda\right)d\lambda}=\frac{\int_{\Delta\lambda_{1}} M_{b}\left(\lambda,T\right)\cdot F_{1}\left(\lambda\right)d\lambda}{\int_{\Delta\lambda_{2}} M_{b}\left(\lambda,T\right)\cdot F_{2}\left(\lambda\right)d\lambda}$ (S11)

This derivation demonstrates that, under the gray-body approximation, the signal ratio $R(T)$ is effectively decoupled from the target emissivity $\varepsilon(\lambda,T)$, depending primarily on the Planck function and the known system spectral responses, $F_{1}(\lambda)$and $F_{2}(\lambda)$. Consequently, for a defined dual-band optical architecture, a physically determined, monotonic mapping function $T=f(R)$ exists. Once this relationship is established via experimental calibration or theoretical calculation, the target temperature $T$ can be reliably retrieved from the measured ratio $R$, thereby effectively mitigating measurement errors arising from indeterminate emissivity within the tested range.

To implement the proposed principle, a bifocal metalens was employed to exert independent and parallel phase control over the optical fields within the operating bands centered at 9.760 ± 0.132 μm and 10.561 ± 0.123 μm. These bands were selected within the long-wave infrared (LWIR) atmospheric window ($8\text{-}14 \mu\text{m}$) to ensure high transmittance in practical scenarios. System calibration was conducted using a standard blackbody radiation source over a temperature range of $100\text{-}450^{^{\circ}}\text{C}$, where a series of dual-band images were acquired under uniform thermal conditions. For each calibration temperature $T_{i}$, the spatially averaged signal ratio $R_{i}=\langle I_{1}\rangle/\langle I_{2}\rangle$ was calculated over the full frame or a region of interest, and the resulting dataset $(R_{i},T_{i})$ was subjected to curve fitting. Due to the intrinsic properties of the Planck function, this relationship exhibits excellent linearity or monotonic nonlinearity within finite temperature ranges, yielding a high-precision calibration function $T=f_{\text{cal}}(R)$. This function implicitly accounts for all practical factors governing the system spectral responses $F_{1}(\lambda)$and $F_{2}(\lambda)$. For target measurement, dual-band images are captured and spatially registered; the local signal ratio $R(x,y)=I_{1}(x,y)/I_{2}(x,y)$ is then computed for each pixel $(x,y)$ and substituted into the calibration function $f_{\text{cal}}$ to directly resolve the corresponding temperature$T(x,y)$, ultimately generating a two-dimensional thermal map that mitigates emissivity-induced uncertainties.

Based on the theory of dual-band ratio pyrometry governed by Planck’s law, we designed a thermal imaging system targeting two infrared bands near 9.6 μm and 10.6 μm. In the experiments, due to the availability of commercial optical filters, the actual passbands were centered at 9.760 ± 0.132 μm and 10.561 ± 0.123 μm, respectively. Combined with a metalens capable of parallel manipulation of these two bands, the system was experimentally implemented. This approach mitigates emissivity-induced uncertainty under the gray-body assumption—a critical source of uncertainty—providing a robust theoretical and technical pathway for miniaturized, high-precision non-contact thermometry in complex environments. Future work may focus on optimizing band selection to enhance robustness against non-gray body behavior, as well as developing advanced algorithms to compensate for environmental reflections and optical aberrations.

**Supplementary Note 3. Discussion on temperature retrieval errors induced by non-gray body behavior**

In the theoretical framework of dual-band ratiometric thermometry, the mathematical accuracy of temperature retrieval is fundamentally predicated on the gray-body assumption, which posits that the target's spectral emissivity remains approximately constant across the operational wavebands ($\varepsilon(\lambda_{1},T)\approx\varepsilon(\lambda_{2},T)$). Under this idealization, the measured signal ratio $R$ effectively cancels the emissivity term, allowing the target temperature $T$ to be retrieved with significantly reduced dependence on indeterminate surface characteristics. However, for materials exhibiting pronounced non-gray radiative characteristics, the spectral emissivity possesses a non-trivial gradient ($\nabla_{\lambda}\varepsilon\neq0$). Consequently, the expression for the signal ratio $R$ evolves to include the emissivity ratio:

$R=\frac{\varepsilon(\lambda_{1},T)}{\varepsilon(\lambda_{2},T)}\cdot\frac{f(\lambda_{1},T)}{f(\lambda_{2},T)}$ (S12)

where $f(\lambda,T)$ represents the system spectral response function governed by Planck’s law. When the ratio $\varepsilon_{1}/\varepsilon_{2}$ deviates from unity, the systematic retrieval error arising from this emissivity mismatch becomes non-negligible.

The quantitative impact of non-gray behavior on thermometric precision is primarily dictated by the morphological evolution of the target’s spectral emissivity curve. Specifically, if the target material exhibits a strong emission peak at $9.6 \mu\text{m}$ or a spectral emissivity that decreases monotonically with wavelength ($\varepsilon_{9.6}>\varepsilon_{10.6}$), the measured signal ratio $R$ will exceed the theoretical expectation of an ideal gray-body model. Because the mapping function between $R$ and $T$ is physically determined and monotonic, this discrepancy leads to a systematic overestimation of the retrieved temperature relative to its true physical value. Conversely, should a strong emission peak occur near 10.6 μm, or if the material displays a pronounced positive spectral slope within the long-wave infrared (LWIR) window ($\varepsilon_{9.6}<\varepsilon_{10.6}$), the signal ratio is attenuated by the emissivity mismatch, resulting in an underestimation of the temperature. Thus, the spectrally selective radiative properties of the target constitute the core physical factor determining both the direction and magnitude of the retrieval error.

Despite the potential interference from non-gray behavior, our system effectively suppresses retrieval bias through a synergistic hardware-software strategy. The dual operational bands, centered at 9.6 μm and 10.6 μm, feature a narrow spectral spacing of approximately 1 μm. This configuration exploits the physical reality that the spectral emissivity of most natural and industrial materials remains relatively flat over such restricted intervals, thereby minimizing the modulation of the ratio signal $R$ by spectral gradients. Simultaneously, both bands are situated within the LWIR atmospheric window, precisely circumventing the dominant absorption features of water vapor and carbon dioxide. This ensures that the radiant intensity captured by the sensor is primarily driven by the target's intrinsic emission, significantly weakening the indirect interference of atmospheric attenuation and ambient noise on the ratiometric model. This physically grounded spectral selection strategy not only enhances system adaptability in complex environments but also provides a robust technical foundation for high-precision, non-contact integrated thermometry platforms.

Experimental validation further confirms the efficacy of this ratiometric strategy. As illustrated in Figure S3, the measured temperatures maintain high linearity with the standard blackbody temperatures across different distances. Despite minor environmental noise, the standard error remains within a narrow margin, demonstrating the robustness of the dual-band model in practical applications.


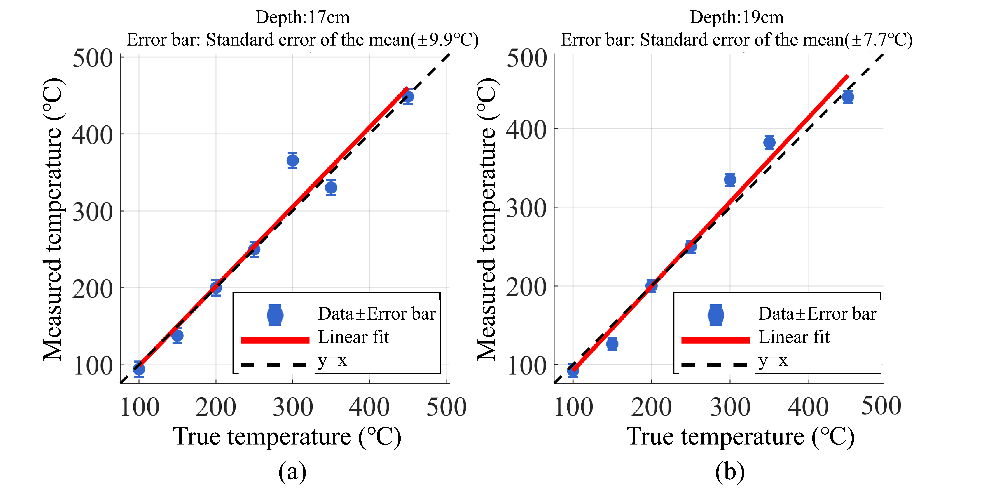


**Figure S3. Temperature measurement error analysis at varying detection depths.** (a) Thermometry results at a target distance of 17 cm, exhibiting a standard error of the mean (SEM) of $\pm9.9$°C; (b) thermometry results at a target distance of 19 cm, with an SEM of $\pm7.7$°C. The blue scatter points with error bars represent the measured values and their corresponding SEM. The red solid line denotes the linear fit, while the black dashed line indicates the ideal reference ($y=x$). These results demonstrate that the system maintains high precision across the temperature range of 100°C to 450°C.
